# Supplementary material for: Distinction between pristine and disorder-perturbed charge density waves in ZrTe3
Source: Nat Commun. 2020 Jan 7;11:98. doi: 10.1038/s41467-019-13813-y (PMC6946692; doi:10.1038/s41467-019-13813-y)
Supplement: Supplementary file 1 — Supplementary Information [file 41467_2019_13813_MOESM1_ESM.pdf]

Supplementary Information for "Distinction between pristine and disorder-perturbed charge density waves in $ZrTe_3$" by Yue *et al.*

1 Supplementary Figures

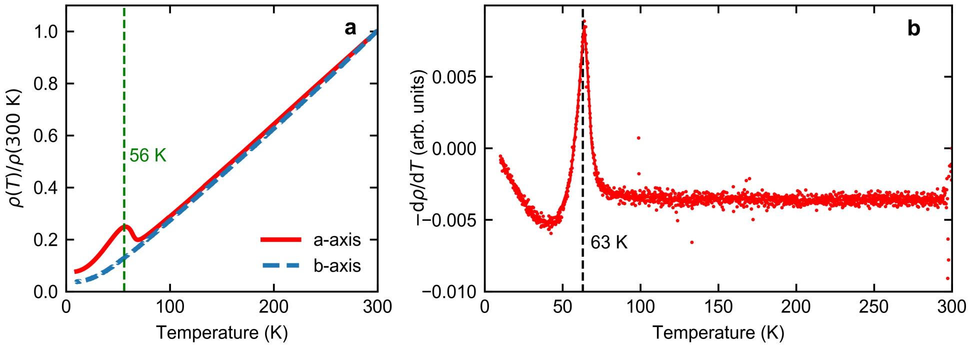

Supplementary Figure 1 Resistivity. (a) Temperature dependence of resistivity measured with electric current running along the crystallographic a and b axes. The data have been normalized at 300 K. (b) The negative value of the temperature derivative of the resistivity along a axis. A maximum is reached at 63 K. Solid line is a guide to the eye.

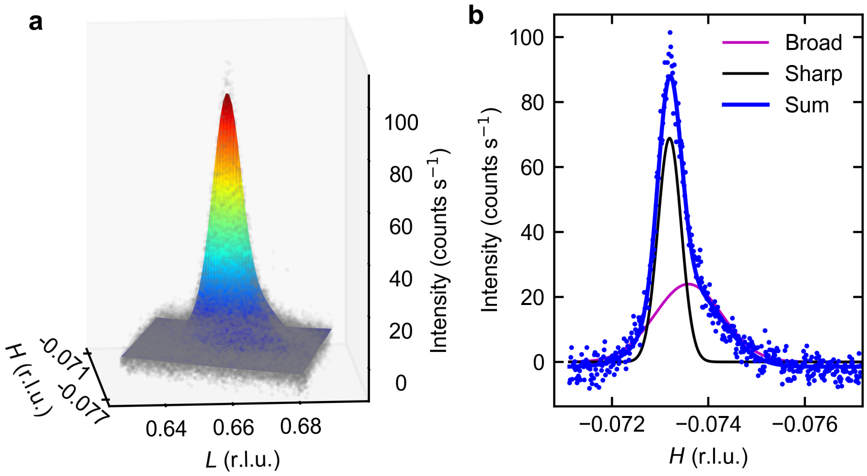

Supplementary Figure 2 Two-dimensional Gaussian fit of diffraction signals. (a) Demonstration of two-dimensional Gaussian fit for the superposed sharp and broad peaks at $T = 62$ K in Fig. 2(a). The translucent grey dots in represent the data points, and the colored surface represents the fit result. (b) A one-dimension cut through the data in (a) along the H direction, at the averaged L position of the two peaks.

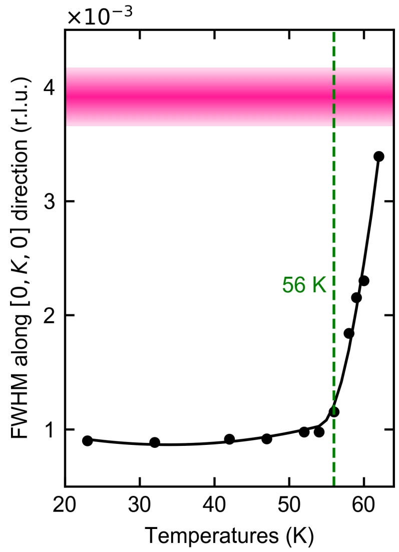

Supplementary Figure 3 Charge correlations along $\mathbf{b}^*$. Temperature evolution of the full width at half maximum (FWHM) of the sharp peak (in Fig. 2 of main text) along $\mathbf{b}^*$. Horizontal shaded stripe indicates the FWHM (and its uncertainty) of the broad peak.

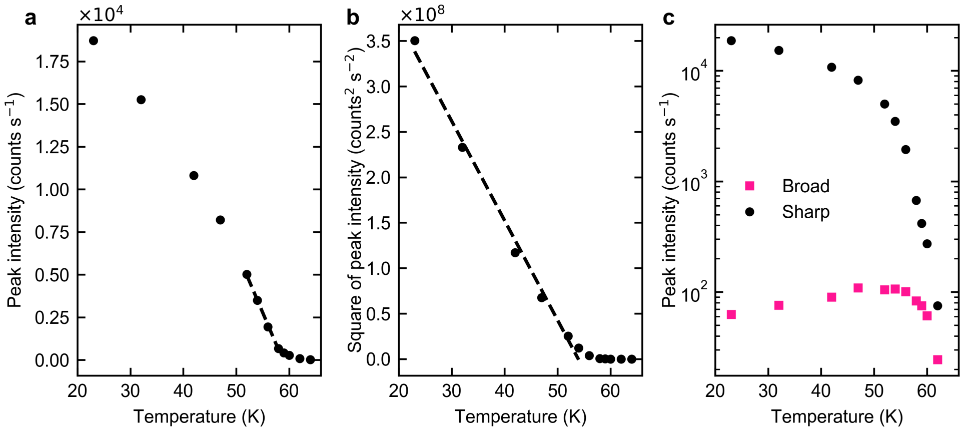

Supplementary Figure 4 The actual transition temperature into long range order. (a) The sharp peak's intensity as a function of temperature. Dashed line represents a linear fit to the data between 52 K and 58 K, resulting in an estimate of the transition temperature of about 59 ± 1 K and a mean-field behavior of the order parameter in the fitted temperature range. (b) The peak intensity squared as a function of temperature. Dashed line represents a linear fit to the data between 23 K and 54 K, resulting in an estimate of the transition temperature of about 54 ± 1 K and a non-mean-field behavior of the order parameter (with critical exponent of about 0.25) in the fitted temperature range. (c) The sharp and broad peak intensities as functions of temperature plotted in log scale.

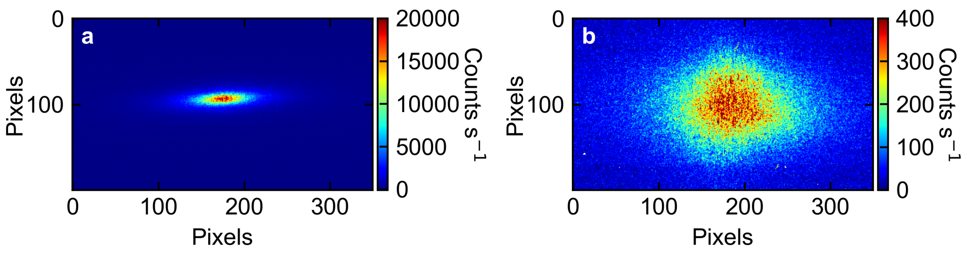

Supplementary Figure 5 Sample quality. Raw FCCD images of the CDW signal of (a) a high-quality crystal at 32 K and (b) a purposely quenched crystal at 27 K. The quench was performed by heating up pristine crystals to 850 °C, followed by cooling in furnace to below 400 °C within one hour.

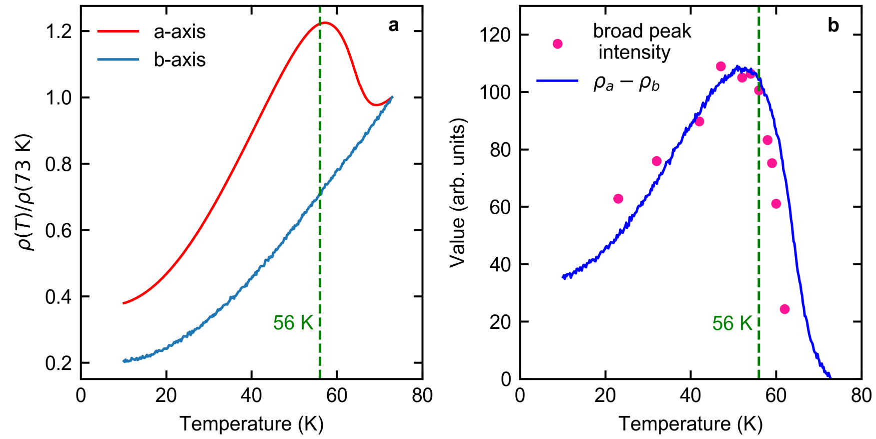

Supplementary Figure 6 Resistivity anomaly and disorder scattering. (a) Temperature dependence of resistivity measured with electric current running along the crystallographic a and b axes, normalized at 73 K. (b) Comparison of the CDW-related resistivity anomaly, extracted from the difference between the two measurements in (a), and the broad peak's intensity in Fig. 2(d) of main text. The two plots match well, suggesting that the resistivity anomaly is caused by enhanced disorder scattering which is a consequence of the self-amplifying FOs (see discussion in main text).

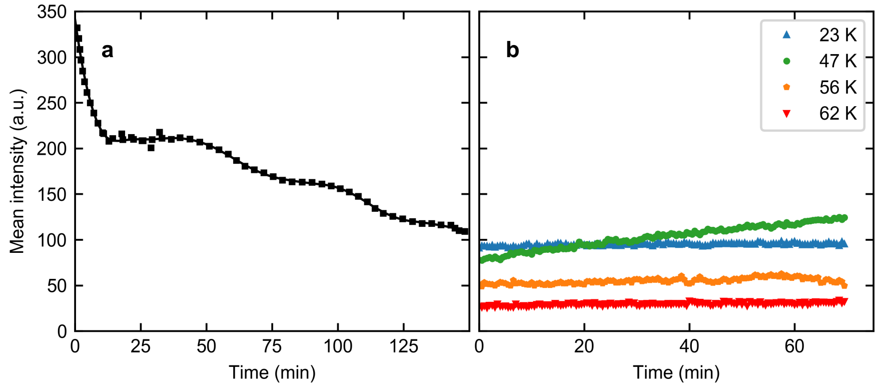

Supplementary Figure 7 Degradation of the sample surface under X-ray. (a) Intensity of CDW diffraction speckles over time after exposing a fresh spot on sample surface which had not been previously illuminated by the X-ray. The measurement was taken at T = 23 K. According to our observations, the intensity decay is related to a degradation of the sample surface, and it does not introduce any noticeable changes in the CDW physics or in the domain distribution as encoded in the speckle patterns, once the intensity has been normalized to correct for the decay. (b) The intensity of diffraction speckles as a function of time for the waterfall plots in Fig. 3(c-f).

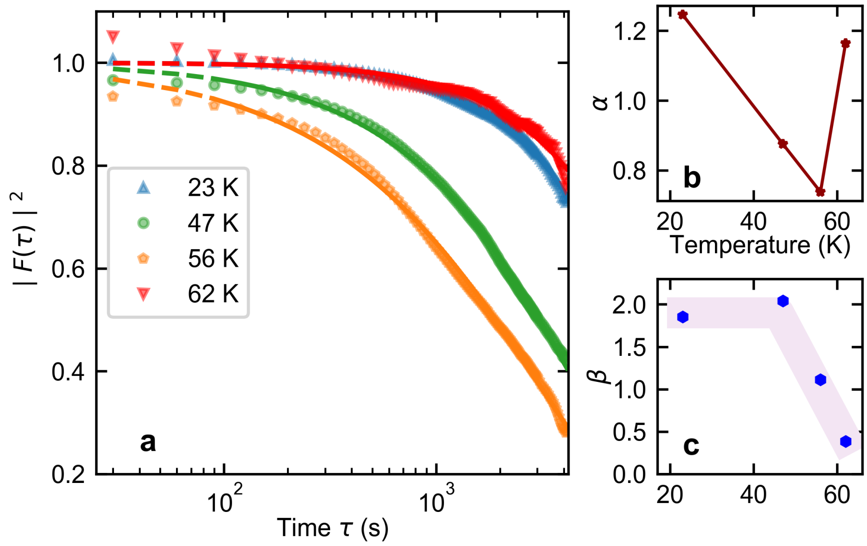

Supplementary Figure 8 Autocorrelation calculation. Autocorrelation of speckle patterns at different temperatures starting from $\tau = 30$ s, along with fit parameters as functions of temperature.

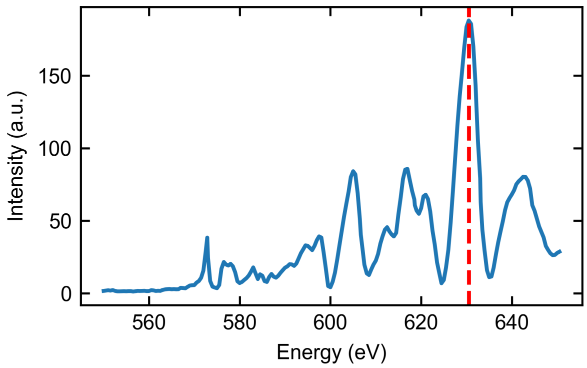

Supplementary Figure 9 Resonant condition. Intensity of CDW diffraction signal as a function of incident X-ray photon energy, measured with momentum-transfer $\mathbf{Q}$ fixed to $(-0.07, 0, 0.67)$ r.l.u. Dashed line marks the X-ray energy of 630 eV, which was used for all the diffraction measurements presented in the manuscript.
